# Specialized Plant Growth Chamber Designs to Study Complex Rhizosphere Interactions

**DOI:** 10.3389/fmicb.2021.625752

**Published:** 2021-03-26

**Authors:** Mon Oo Yee, Peter Kim, Yifan Li, Anup K. Singh, Trent R. Northen, Romy Chakraborty

**Affiliations:** ^1^Climate and Ecosystem Sciences, Earth and Environmental Sciences Area, Lawrence Berkeley National Laboratory, Berkeley, CA, United States; ^2^CBRN Defense and Energy Technologies, Sandia National Laboratories, Livermore, CA, United States; ^3^The DOE Joint Genome Institute, Lawrence Berkeley National Laboratory, Berkeley, CA, United States

**Keywords:** rhizosphere, interactions, plant growth chamber, soil, chamber design

## Abstract

The rhizosphere is a dynamic ecosystem shaped by complex interactions between plant roots, soil, microbial communities and other micro- and macro-fauna. Although studied for decades, critical gaps exist in the study of plant roots, the rhizosphere microbiome and the soil system surrounding roots, partly due to the challenges associated with measuring and parsing these spatiotemporal interactions in complex heterogeneous systems such as soil. To overcome the challenges associated with *in situ* study of rhizosphere interactions, specialized plant growth chamber systems have been developed that mimic the natural growth environment. This review discusses the currently available lab-based systems ranging from widely known rhizotrons to other emerging devices designed to allow continuous monitoring and non-destructive sampling of the rhizosphere ecosystems in real-time throughout the developmental stages of a plant. We categorize them based on the major rhizosphere processes it addresses and identify their unique challenges as well as advantages. We find that while some design elements are shared among different systems (e.g., size exclusion membranes), most of the systems are bespoke and speaks to the intricacies and specialization involved in unraveling the details of rhizosphere processes. We also discuss what we describe as the next generation of growth chamber employing the latest technology as well as the current barriers they face. We conclude with a perspective on the current knowledge gaps in the rhizosphere which can be filled by innovative chamber designs.

## Introduction

Roots are not only vital for anchorage and for acquisition of water and nutrients from the soil, but are also engaged in complex physical and chemical interactions with the soil. Plant roots release approximately 11–40% of their photosynthetically fixed carbon, commonly known as root exudates, into the soil (Sasse et al., 2018; Zhalnina et al., 2018a). Root exudates and mucilage act as nutrient sources and as signaling molecules for soil microorganisms, thus shaping the microbial community in the immediate vicinity of the root system (Venturi and Keel, 2016). In turn, microbial processes promote plant growth by aiding in nutrient acquisition, plant growth hormone production and bio-control of plant pathogens (Afzal et al., 2019). The physicochemical characteristics of the surrounding soil are also affected by interactions between roots and the microbial community. This interplay between the different rhizosphere components is affected by spatio-temporal processes, which culminates in dynamic feedback loops that maintain the complex rhizosphere environment with physical, chemical and biological gradients that are distinct from the bulk soil (Six et al., 2004; Koebernick et al., 2017). Understanding these intricate rhizosphere relationships is vital in devising strategies to increase plant productivity and comprehend localized biogeochemical processes.

In many rhizosphere studies, the use of pots and containers is predominant as it allows the plants to be cultivated under controlled conditions and at low cost. Compared to field studies, growth of plants in defined spaces (e.g., pots) also offers advantages in ease of handling, monitoring and sampling (Neumann et al., 2009). Much of what we know of the rhizosphere microbiome has resulted from such pot-grown plants. However, since the rhizosphere and roots are still out of view in the soil, destructive sampling of the root is required prior to analysis. Destructive sampling may result in the loss of three-dimensional (3D) spatial information on rhizosphere processes over time, which is increasingly being recognized as a critical parameter.

On the other hand, soil free techniques such as hydroponics and aeroponics can provide visual access to the rhizosphere circumventing the need for destructive sampling. Other alternatives are gel-based substrates which can maintain rhizosphere transparency as well as the 3D architecture of roots and have been applied successfully in high throughput imaging, phenotyping and trait mapping platforms (Iyer-Pascuzzi et al., 2010; Topp et al., 2013). Nonetheless, the root phenotype and traits of plants grown under soil-free conditions are known to differ from those of soil-grown plants (Kuijken et al., 2015). These soil substitutes do not also accurately simulate the heterogeneous nature of soil aggregates, thus complicating extrapolations for field relevance. Sophisticated imaging approaches such as magnetic resonance imaging (Metzner et al., 2014; Popova et al., 2016; van Dusschoten et al., 2016) and X-ray computer tomography (Mooney et al., 2012; Helliwell et al., 2013) can be used to analyze root systems in the soil with minimal disturbance but they are low throughput, expensive and may not be easily accessible (Oburger and Schmidt, 2016; Morris et al., 2017). It is apparent that structural changes in design catered to solving specific challenges in the rhizosphere are indeed necessary.

To overcome these challenges relating to the rhizosphere in soil, specialized plant growth chamber systems have been designed, and successful implementation has led to multiple variations of similar designs. These specialized systems often have a visible rhizosphere which enables coupling with other technologies thereby increasing the breadth of experimental techniques applicable to the rhizosphere system. This review discusses representative growth chamber systems designed to study major rhizosphere processes and interactions in soil. Growth platforms resembling conventional containers such as pots and tubes are not covered. Specifically, the reviewed growth systems are selected based on the following criteria: (1) the growth chamber is amenable for use with soil/soil-like substrates (e.g., vermiculite or sand) and therefore, hydroponics, aeroponics and agar/gel-based systems are not discussed except in microfluidic-based platforms, (2) it is built with the intention to maintain growth of the plant and has architectural features distinct from conventional pots, and lastly (3) it is able to be set up in a laboratory; i.e., field measurement systems and observation platforms are not included. For instance, a minirhizotron, consisting of a camera mounted in a glass tube submerged in the soil which provides non-destructive root imaging over time (Taylor et al., 1990) will not be discussed as it is out of the scope of this review. Through our assessment of lab-based chamber systems, we identify unique advantages and challenges associated with each system (Table 1). We hope that future fabrication designs can benefit and improve on designs that work well. Lastly, we offer our perspectives on areas in which technological advances are needed to fill current knowledge gaps.

**TABLE 1:** Key attributes of different growth chambers designed to study rhizosphere processes and interactions.

Growth chamber setup	Basic design principles	Advantages	Disadvantages	Experimental scale	Tested rhizosphere processes	References
Rhizotron/rhizobox setup	- Chamber built with two sheets often made of PVC or acrylic, of which at least one sheet is transparent and/or removable. - Many chamber designs are based of this basic set up	- Versatile and easy set up. - Allows visualization of the rhizosphere. - Can be coupled to many visualization techniques.	- Information limited to 2D plane. - Loss of information on roots occluded by soil particles.	cm to m	All major rhizosphere processes possible.	Devienne-Barret et al., 2006; Neumann et al., 2009; Bontpart et al., 2020
Rhizobox with side-compartment	- A side chamber is built into a basic rhizobox connected via a controlled aperture. - Rhizosphere visualization is on the side chamber.	- Allows isolation of individual roots via controlled root growth through the aperture. - Easy differentiation of old vs. new roots	- Root growth into side compartment only controlled via timing of aperture opening. - Loss of information on roots occluded by soil particles.	cm	Bacterial interactions	Jaeger et al., 1999; Nuccio et al., 2020
Vertical root mat chambers	- Root growth is restricted from the soil through a size-selected membrane; root hairs and solutes move freely through the membrane. - Can maintain full plant growth or act as secondary container for root only growth	- Allows visualization of the whole root system.	- Unnatural root growth in complete 2D plane	mm to cm	Exudate collection, Nematodal interactions	Oburger et al., 2013; Dinh et al., 2014
Horizontal root mat in rhizobox	- Particularly used in root exudate collection. - Root growth is restricted by membrane at the bottom of rhizobox; root hairs and solutes move freely through the membrane	- Possibility of root exudates collection into soil or liquid substrate. - Possibility of root growth in soil substrate	- Unnatural rhizosphere environments in high density root mat.- Tangled roots and loss of exudate profiles from individual roots	mm to cm	Exudate collection, Physicochemical gradients in the soil	Chaignon et al., 2002; Chaignon and Hinsinger, 2003
Mycorrhizal compartments	- Rhizobox compartments separated by membranes to restrict movement of roots but not hyphae of mycorrhizal fungi or solutes. - An additional wire net may be placed between compartments to create air gap to restrict solute movement	- Long range (cm) foraging capabilities and connectivity of mycorrhizal hyphae	- Visualization of mycorrhizal hyphae not possible	cm	Fungal interactions	Tanaka and Yano, 2005; Kaiser et al., 2015; Wang et al., 2016
Split-root systems	- A physical barrier separates the roots into generally two compartments under different conditions. - Developed roots may be manually split into the compartments or new roots may be directed to grow into the different compartments, often achieved after excising parts of the root	- Enables investigations of the systemic response of plants. - Applicable in non-specialized containers such as pots	- Root damage during split-root transplant.- Cut roots show lower survival rates	cm	Systemic response of plants to rhizosphere processes	Agapit et al., 2020; Saiz-Fernández et al., 2021
Nylon soil pouches	- Nylon membranes often made into bags/pouches restrict root growth. - Applicable in conventional pots as well as specialized rhizoboxes	- Accessible and easy separation of root-free soil from the rhizosphere.	- Over-estimation of rhizosphere range.	cm	Bacterial interactions	Yevdokimov et al., 2006; Wei et al., 2019
Microfluidic chambers	- 3D fabricated flow-through device with seedling port and microchannel for primary root growth	- Allows analysis of microscale processes with high spatiotemporal resolution. - Precise control of the reproducible conditions utilizing the laminar flow and automated fluidic operations. - Well integrated with conventional imaging techniques.- Rapid prototype testing	- Small size limits choice of plants and testing time frame to young seedling.- Only hydroponics systems to date.	mm	Major rhizosphere processes in hydroponic conditions	Grossmann et al., 2011; Stanley et al., 2016
EcoPODs	- Enclosed pilot scale ecosystem chambers with multiple built-in equipment and sensors	- Manipulation of various aspects of environmental conditions above and below ground of the plant. - Bridges the gap between lab scale studies to field studies	- Not easily accessible. - Significant financial investment involved. - Requires dedicated maintenance	cm to m	All major rhizosphere processes possible	Ke et al., 2020
EcoFABs	- 3D fabricated flow-through devices designed for the development of model rhizosphere ecosystems	- 3D fabrication allows easy adaptation and modification to the system. - Standardized protocols increases reproducibility. - Rapid prototype testing	- Small size limits choice of plants and testing time frame.- Roots limited to a plane	mm to cm	Microbial interactions demonstrated so far	Gao et al., 2018; Zengler et al., 2019

*The corresponding schematic images for the different chambers are illustrated in Figure 2.*

**FIGURE 1:**
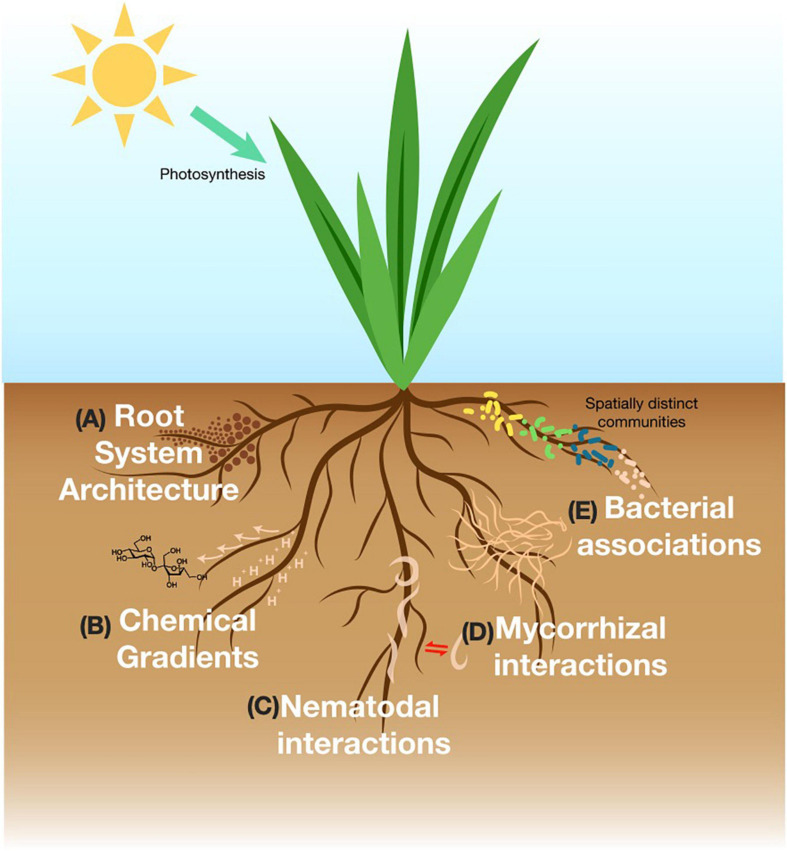
Representative figure of major rhizosphere processes in the soil discussed in this review. **(A)** Root system architecture is concerned with structural features of the root and responds to with environmental stimuli. **(B)** The rhizosphere produces photosynthetically fixed carbon that exudes into the soil and influences soil physicochemical gradients. **(C)** Free-living or parasitic nematodes interact with the rhizosphere via signaling interactions. **(D)** Mycorrhizal fungi create intimate relationships with the roots and engage in nutrient exchange. **(E)** Bacterial composition is distinct upon different parts, age, type of the roots.

**FIGURE 2:**
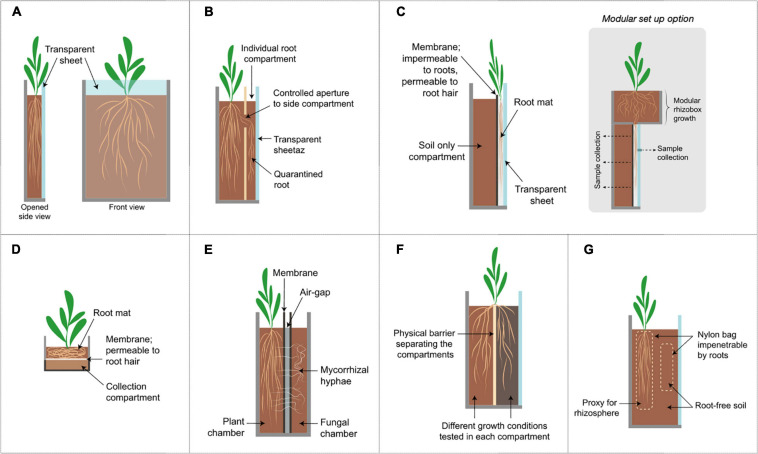
Schematic diagrams of representative growth chamber designs. Further description and characteristics are listed in Table 1. **(A)** Rhizotron/Rhizobox set up, **(B)** Rhizobox with side-compartment, **(C)** vertical root mat chambers; a modular option is show where the plant can be pre-grown in a separate compartment and transplanted afterward onto the main examination chamber, inset shows a modular set up option, **(D)** horizontal root mat in rhizobox, **(E)** Mycorrhizal compartments, **(F)** split-root systems shown here in a rhizobox set up; **(G)** Nylon bag to separate root and root-free soil; roots may be restricted in the bag or the soil may be protected from root penetration by the bag.

## Specialized Chambers to Study Major Rhizosphere Interactions

In studying rhizosphere processes, the myriad of complex interactions among members of the rhizosphere are often dissected to two interacting variables such as root-and-soil or root-and-microbes, etc. Each of these interactions inherently operates under distinct parameters and requires specifically designed platforms to effectively answer different research questions. This review is structured in a way that first describes each rhizosphere process briefly and then reports on the specific growth chamber systems designed to facilitate experiments for answering related research questions. The major rhizosphere processes discussed below include root system architecture, physicochemical gradients in the soil, exudation patterns by the roots and interactions between roots and nematodes, fungi or bacteria.

### Investigating Root System Architecture

Root system architecture (RSA) encompasses structural features that provide spatial configuration such as root length, width, spread and number (Khan et al., 2016; Figure 1) and is an important rhizosphere parameter in regulating soil porosity, and nutrient and water uptake efficiency by plants (Helliwell et al., 2017; Fang et al., 2019). Plants have been observed to “sense” and direct root growth toward nutrient sources in soil, and the RSA of a plant exhibits great malleability in response to environmental stimuli (changes in nutrients, pH, soil moisture, and temperature) which in turn, influences microbial communities (Bao et al., 2014; Saleem et al., 2018). For instance, bean plants grew deeper roots under drought conditions to enhance water foraging capabilities while low phosphate (P) conditions stimulated the formation of dense lateral roots involved in P uptake from upper soil layers (Ho et al., 2005). Given that most soils are heterogenous, understanding the RSA of plants becomes critical in improving resource use efficiency and agricultural yields (Ingram and Malamy, 2010; Khan et al., 2016). Often, RSA in pot-grown plants is investigated by excising the roots via mechanical means such as root washing or blowing with compressed air (Judd et al., 2015). These methods are, however, time-consuming, cause inevitable damage of fine root hairs and result in loss of spatial and temporal information (Judd et al., 2015).

An appealing alternative for studying RSA is the use of rhizotrons. Rhizotrons were initially constructed as underground facilities designed for viewing and measuring roots in the field (Klepper and Kaspar, 1994). In the lab, the rhizotron implies a chamber constructed using two vertical sheets with at least one or both of the sheets being transparent and/or removable (Figure 2A). This allows repeated visual inspections of individual roots; a feature unachievable with destructive sampling. In some cases, the word “rhizobox” is used for a similar set up although this was first introduced in as compartmentalized systems to separate the root and soil compartments (Kuchenbuch and Jungk, 1982). Rhizotrons/rhizoboxes are often constructed with PVC or acrylic materials and come in many sizes to accommodate different plants with soil or soil-less substrates (Neumann et al., 2009). Root growth and morphology in the rhizotron can be tracked by a variety of methods ranging from manual tracing onto a plastic sheet, using handheld or flatbed scanners to fully automated time-lapse imaging camera systems (Mohamed et al., 2017). Data can be subsequently analyzed with a wide range of software packages (Kuijken et al., 2015). Affordable and robust RSA imaging platforms using rhizotrons have also been developed for increased accessibility in low-income countries (Bontpart et al., 2020).

The versatile construction of a rhizotron design for RSA studies has inspired many variations. For instance, ara-rhizotrons were designed to enable the study of 3D canopy competition with simultaneous root growth observation in an Arabidopsis plant population (Devienne-Barret et al., 2006). The horizontal and radial design of Horhizotron^TM^ and mini-Horhizotron consisting of transparent quadrants attached to a central chamber were developed to study lateral growth of roots in a semi-3D space and to perform post-transplant assessment (Wright and Wright, 2004; Judd et al., 2014). The separated quadrants can also be used with different soil substrates simultaneously to study substrate effects on root growth (Wright and Wright, 2004). A rhizotron fitted with water-tight gasket seals has also been used successfully to investigate the RSA of plants under water-logged conditions (Busch et al., 2006).

Despite the continuous real-time visual read-out, most rhizotron designs suffer from inevitable loss of information from roots occluded by soil particles. The GLO-Roots system overcomes this by imaging from both sides of the rhizotron while using bioluminescent roots to create higher contrast against the soil, enabling quantitative studies on RSA (Rellán-Álvarez et al., 2015). Following advances in engineering and device fabrication, more rhizotron variants adapted to specific plant growth conditions can be envisioned.

### Mapping Physicochemical Gradients in the Rhizosphere

In a typical topsoil, approximately half is composed of solid minerals and organic matter while the rest is a fluctuating composition of water and gas filled spaces influenced by environmental conditions and uptake/release of solutes from plants (O’Donnell et al., 2007). Changes in gaseous and hydrologic parameters, such as ions, O_2_ and moisture among others, create a spatially complex environment that influences microbial communities and overall plant health. These physicochemical fluxes are heterogeneously distributed along roots and vary with root types and zones (Neumann et al., 2009). Often, they exist as gradients in the rhizosphere (Kuzyakov and Razavi, 2019), thus emphasizing the need for non-destructive sampling in order to accurately capture processes occurring at biologically relevant times and scales.

Rhizotron chambers with a visually accessible rhizosphere allows *in situ* and continuous mapping of these gradients in the soil through the use of different types of imaging methods. For instance, photoluminescence-based optical sensors enable *in situ*, repeated detection of small molecule analytes in addition to pH (Blossfeld et al., 2010), O_2_ (Frederiksen and Glud, 2006) and NH_4_ (Santner et al., 2015). Methods like zymography to detect enzyme activity (Spohn et al., 2013) and diffusive gradients in thin film (Santner et al., 2012; Valentinuzzi et al., 2015) can be used to map solute concentrations in the soil down to sub-mm scales with high spatial resolution more realistically than traditional destructive approaches. For example, transport and distribution of water in the rhizosphere soil has been imaged on both 2D and 3D planes by coupling a rhizotron with neutron radiography and tomography, respectively (Esser et al., 2010) and showed varying moisture gradients along the root system with higher water uptake at the rhizosphere compared to bulk soil. On the other hand, if the rhizotron slabs are thin enough (∼4 mm), even simple imaging solutions based on light transmission can be set up to capture water uptake by roots in sand (Garrigues et al., 2006). Despite trade-offs in method sensitivity between these two studies, a rhizotron set up is critical in both designs and illustrates its adaptability to multiple equipment.

### Characterizing Root Exudates

Roots exude a substantial amount of photosynthetically fixed organic carbon into the soil consisting of a wide variety of compounds such as sugars, organic acids, and primary and secondary metabolites (Sasse et al., 2018; de la Fuente Cantó et al., 2020). Together with mucilage and border cells (which are mainly expelled from root tips), root exudates provide a major source of nutrients for the rhizosphere microbiome (Figure 1). Root exudation is regulated under genetic control (i.e., genotype, root type and developmental stage) (Canarini et al., 2019) as well as in response to environmental conditions in the soil such as nutrient limitations or increase in toxicity (van Dam and Bouwmeester, 2016). Exudate patterns are also recognized as one of the strongest drivers shaping the rhizosphere microbiome (Dessaux et al., 2016; Zhalnina et al., 2018b; de la Fuente Cantó et al., 2020). As a central player in the rhizosphere ecosystem, it is imperative to understand root exudation patterns to unravel subsequent impacts to the surrounding soil and microbial community.

Improvements in analytical instrumentation have made it possible to move from targeted to untargeted explorations with mass spectrometry to create root exudate fingerprints in its entire complexity (Oburger and Schmidt, 2016). Regardless, the impact of such techniques relies partly on our exudate sampling techniques. Detection of exudates in real-time is difficult due to rapid biotransformation and sorption to the soil matrix. As such, common collection methods rely on root washing in hydroponic systems to overcome complications in the soil matrix and preserve native exudation profiles. However, a comparison between a soil-based collection method and hydroponic methods showed varied responses particularly in amino acid exudation although the underlying cause was not elucidated (Oburger et al., 2013). It is possible that the differing growth conditions between hydroponics and soil, which include differences in gas concentrations, mechanical impedance and microbial spatial composition, can elicit differing root exudation responses to the same environmental stimuli.

Rhizoboxes offer the advantage of localized sampling in soil using sorption media such as paper and membrane filters, compound specific ion exchange binding resin or micro-suction cups placed closed to root zones of interest to collect exudates (Kamh et al., 1999; Neumann et al., 2009, 2014). Moreover, in a rhizobox fitted at the bottom with a porous root-impenetrable membrane, a root mat is allowed to be formed which is then further transferred onto a collection compartment (Figure 2D; Chaignon et al., 2002; Chaignon and Hinsinger, 2003). The collection compartment containing soil could then be cut into thin slices (1–3 mm) parallel to the membrane to represent differing distances from the rhizosphere (Neumann et al., 2009). While this approach can be used to investigate exudate release and sorption under soil conditions, the root mat growth generalizes exudate production in terms of the whole root system and occludes spatial exudation patterns. In a hybrid set up by Oburger et al. (2013), the rhizobox is transplanted to a second specialized rhizobox for continued vertical root growth. This specialized rhizobox consists of a nylon membrane (30 μm pore size) close to the transparent side to restrict root growth into the soil except for root hairs (Figure 2C). This creates a vertical flat root mat onto which localized exudate samples can be collected. A comparison of this novel set up to conventional collection methods showed that amino acid exudation rates were most varied among the different methods (Oburger et al., 2013), further highlighting the need for specialized chambers.

Nonetheless, successful implementation of these chambers is still limited to fast-growing plants which can form active root mats. The high density of root mats could also lead to unnatural root exudate levels and an overestimation of rhizosphere effects. In addition, care has to be given to the choice of membrane as selective sorption of certain root exudates onto the membrane may also occur (Neumann et al., 2009).

### Investigating the Biology and Ecology of Rhizosphere Nematodes

Free-living nematodes are ubiquitous in the soil. They are beneficial to the plants by playing a role in nutrient cycling and in defense against insects and microbial infections through signaling interactions with the roots (Rasmann et al., 2005; Manosalva et al., 2015; Figure 1). Conversely, infections by parasitic nematodes in the roots increase the plant’s susceptibility to stress and other pathogenic bacteria, fungi, and viruses creating major losses in crop productivity (Powell, 1971; O’Callaghan et al., 2018). With an impending rise in nematode infections due to climate change, understanding nematode behavior and interactions in the rhizosphere becomes important to develop appropriate biocontrol methods to ensure long term food security (O’Callaghan et al., 2018).

Traditional nematode studies are performed in petri dishes with agar or culture media (Dinh et al., 2014; O’Callaghan et al., 2018). However, these substrates do not accurately emulate the physical textures and heterogeneity of soil and create homogenous solute and temperature gradients which could impact nematode behavior and interactions with the roots (Lockery et al., 2008). Indeed, nematode motility speed and dispersal decreased in substrates more closely mimicking sand (Hapca et al., 2007). On the other hand, studying nematode behavior in the soil is a difficult endeavor as its near-transparent body and small size makes it almost indistinguishable from soil particles. Cross-sectioning and staining infected roots make it possible for nematode visualization but they are destructive and provide only static snapshots of cellular changes or nematode behavior during infections (Dinh et al., 2014).

On the other hand, microscopy rhizosphere chambers provide non-invasive detection and observation of nematode activity in the rhizosphere (Froelich et al., 2011; Kooliyottil et al., 2017). The roots in these chambers grow between a glass slide and a nylon membrane (unknown pore size) (Figure 2C). The membrane restricts movement of roots except root hairs into the soil while the transparent glass enables microscopy of the roots at high resolution (Froelich et al., 2011). Coupled with fluorescently stained nematodes, microscopy rhizosphere chambers allowed for non-destructive *in situ* observations of nematode infection in its host species over the entire life of the parasite (Dinh et al., 2014; Kooliyottil et al., 2016).

Nonetheless, staining nematodes is an additional challenge as nematode cuticles are impermeable to stains (O’Callaghan et al., 2018). This can, however, be alleviated by using advanced imaging technologies which eliminates the need for staining. A recent study demonstrated live screening of nematode-root interactions in a transparent soil-like substrate through the use of label-free light sheet imaging termed Biospeckle Selective Plane Illumination Microscopy (BSPIM) coupled with Confocal Laser Scanning Microscopy (Downie et al., 2014; O’Callaghan et al., 2018). Using this set up, researchers were able to monitor roots for nematode activity at high resolution and suggest its possible use in rapid testing of chemical control agents against parasitic nematodes in soil-like conditions (O’Callaghan et al., 2018).

### Investigating Soil Fungal Communities

Fungal communities in the rhizosphere are involved in the degradation of organic matter in the soil and subsequent nutrient turnover affecting plant health as well as the microbial community (Buée et al., 2009). Fungal biomass often reaches a third of total microbial biomass carbon (Joergensen, 2000) and almost all terrestrial plants are able to form symbiotic associations with mycorrhizal fungi (Van Der Heijden et al., 2016; Figure 1). The majority of these associations are with arbuscular mycorrhiza fungi (AMF) (Smith and Read, 2008) which penetrate into root cortex cells to form highly branched structures (Harrison, 2005). The investment of photosynthetic carbon by plants to AMF is rewarded with increased nutrient availability made possible by the extended hyphal network in the soil. For instance, up to 90% of phosphorus uptake in plants can be contributed by symbiosis with AMF (Ferrol et al., 2019). AMF networks in the soil also influence water retention and soil aggregation further impacting plant growth (Augé, 2004). Moreover, next-generation sequencing technologies and advances in imaging techniques have greatly improved our knowledge on the taxonomical and functional properties of fungal communities in the rhizosphere (Oburger and Schmidt, 2016). However, these methods are optimized for fine scale (millimeter) analysis and are not capable of assessing the foraging capabilities of hyphal networks which can span across centimeter to meter scales.

Toward this end, several researchers have used compartment setups with physical barriers created by 20–37 μm nylon membranes (Figure 2E) which restrict movement of roots but not mycorrhizal fungi. This separation creates root-free and plant-free soil compartments connected only by mycorrhizal fungi to examine the transport of various compounds across these compartments. Using this set up, the importance of mycorrhizal fungi in the flow of different elements such as carbon (Kaiser et al., 2015), nitrogen (Tanaka and Yano, 2005) and phosphorus (Wang et al., 2016) between plants, soil and microbes over centimeter distances have been validated. Repeated disruption of the hyphal connections also led to a decreased resistance in plants to drought stress (Zou et al., 2015). The membranes can also be placed horizontally to create different depth gradients to investigate hyphal contributions to water uptake (Ruiz-Lozano and Azcón, 1995). In some studies, an additional 1.5–3 mm air gap is created between two membranes with a wire net to restrict solute movement between two chambers (Tanaka and Yano, 2005; Zhang et al., 2010; Koegel et al., 2013; Figure 2E). A common feature of these set ups is the size-exclusion membranes which proved to be critical in distinguishing fungal hyphae processes in the rhizosphere soil.

In addition to AMF interactions, a split root set up, which separates the roots of one plant into halves, can be introduced to investigate the systemic response of plants (Figure 2F; Vierheilig et al., 2000). In essence, the split-root system directs the growth of the roots to generally two different growth conditions and enables the investigation of whether a local stimuli (microbial interactions, nutrient limitations, etc.) have a local or global response which can be observed at the root or shoot level (Agapit et al., 2020). Split-root systems are widely studied (Larrainzar et al., 2014; Saiz-Fernández et al., 2021) and have been adapted to rhizoboxes (Zhu and Yao, 2004; Mitchell et al., 2018) as well as to pots and tubes (Kosslak and Bohlool, 1984; Marschner and Baumann, 2003).

### Characterizing Bacterial Interactions

In the rhizosphere, plants host a wide diversity of bacteria on the surface of the root (epiphytes) as well as within roots in the vascular tissue (endophytes). Due to its abundance and importance, the bacterial community in the rhizosphere is perhaps the most widely studied among other microbial members in the rhizosphere ecosystem. While the study of endophytic bacteria requires inevitable destructive sampling due to its localization, several non-destructive approaches have been developed to study microbes inhabiting the rhizoplane.

One of the most widely studied plant-microbe interactions in the rhizosphere is that of the symbiotic relationship between legumes and rhizobia (Hirsch et al., 2001). Once a potential nodule forming bacteria is isolated, it is often required to authenticate its nodule forming phenotype by inoculating on host plants. However, conventional methods such as the use of soil pouches do not allow long term incubation, while “Leonard jars,” consisting of two stacked glass jars forming the top soil layer and the bottom nutrient solution layer, can be expensive and time consuming (Yates et al., 2016). A recent study challenges this by describing the use of clear plastic CD cases as mini-rhizotrons with potential for use in phenotyping root traits such as legume formation, and demonstrated innovation that democratizes research opportunities in rhizosphere research (Cassidy et al., 2020).

Other microbial interactions in the rhizosphere, however, may not result in visible changes to the root system and often rely on next-generation omics technologies. As such, physical separation of the rhizosphere from the bulk soil becomes paramount in elucidating changes to microbial community and interactions. One approach to this end is the use of nylon bags with differing pore sizes (10–50 μm) (Figure 2G). The nylon bag restricts the movement of roots and the soil inside the bag is then regarded as the rhizosphere soil to compare against the surrounding root-free bulk soil (Yevdokimov et al., 2006; Shrestha et al., 2010; Nie et al., 2015). Developing further on this concept, Wei et al. (2019) designed a specialized rhizobox that allowed repeated non-destructive sampling by adding individual nylon bags of root-free soil surrounding the root compartment which are then used as a proxy for the rhizosphere (Wei et al., 2019).

These methods allowed easy distinction of the rhizosphere and the bulk soil but, we now know that the rhizosphere community is not only distinct from the bulk soil but also varies with type, part and age of the root, largely as a consequence of varying root exudation patterns (Sasse et al., 2018). Studying this phenomenon *in situ* in the soil requires separation of desired roots from others without disturbance to plant growth or soil. To address this, researchers have used a modified rhizobox design with a side compartment to regulate root growth and quarantine specific roots from the main plant chamber (Figure 2B). This additionally creates easy distinction between old and new roots and allows testing on specific quarantined roots despite plant age. A study using this set up showed specific microbial chemotaxis toward different exudates (sucrose or tryptophan) on an individual root (Jaeger et al., 1999) whereas another showed spatial and temporal regulation of niche differentiation in microbial rhizosphere guilds (Nuccio et al., 2020). Similar physical perturbations to regulate root growth in response to microbial stimuli have also been applied in the microscale and are explored in the next section.

## Next Generation of Plant Growth Chambers

Our assessment of the major growth chambers showed that most of the systems applied share similarities in basic structural components such as in the use of two parallel sheets in rhizobox-based devices. While these growth chambers brought many of the rhizosphere processes to light, limitations do exist. One limitation is with the scale of applicability. Most of these growth systems are mesoscale and can easily reproduce pot-scale studies (Devienne-Barret et al., 2006) but may not be easily translatable to interactions occurring at the microscale nor recapitulate processes occurring at field-relevant scale. The next section describes advances in technology resulting in a new wave of unique devices making use of microfluidic processes and fabricated ecosystems which are specifically made to investigate specific rhizosphere processes.

### Microfluidic Chambers

**FIGURE 3:**
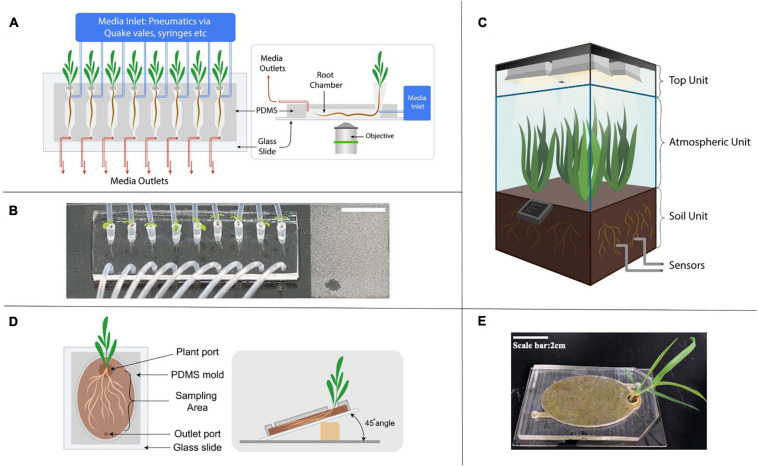
Next generation of growth chamber systems developed to study rhizosphere interactions. **(A)** Schematic diagram of a representative microfluidic device in studying root response to environmental stimuli. The media inlet is controlled by various pneumatic methods such as syringes and automated Quake-valve system. **(B)** An image of a RootChip, demonstrating the increased throughput by the parallel growth of 8 Arabidopsis seedlings on a single device (a figure by Massalha et al., 2017). **(C)** Schematic diagram of an EcoPOD showing three dedicated units with different features; sensors monitor operational parameters closely and the central hub located at the base of the atmospheric unit controls the EcoPOD. **(D)** Schematic diagram of an EcoFAB with soil sealed to a glass slide. **(E)** An image of the EcoFAB bonded to glass slide with *Brachypodium distachyon* grown in soil.

A complex web of biochemical processes and interactions occur in microscale dimensions in the rhizosphere. Having the ability to interrogate and manipulate these microscale processes and environmental conditions with high spatiotemporal resolution will elucidate mechanistic understanding of the processes. Microfluidics has proven to be a powerful approach to minimize reagent usage and to automate the often-repetitive steps. The microscale of the channels also allows precise control of reproducible conditions utilizing the laminar flow and automated fluidic operations (Figures 3A,B). In addition, the microfluidic devices are well integrated with conventional imaging techniques by using a glass slide or coverslip as a substrate bonded with polydimethylsiloxane (PDMS). These characteristics, as well as the ability to rapidly prototype and reproducibly manufacture using soft lithography technique, have enabled new ways of interrogating and studying the rhizosphere environment in a reproducible manner.

Many of the microfluidic devices used for studying the rhizosphere share a similar design concept (Khan et al., 2019). They have an opening port, sometimes with pipette tips inserted into the PDMS body where the seed of the seedling rests and a microchannel where the primary root grows into. The dimension of the channel depends on the type and age of the plant. For example, an *Arabidopsis thaliana’s* seedling is typically grown in a microfluidic device up to 10 days, with chamber dimension around 150 to 200 μm in height, whereas the *Brachypodium distachyon* seedling chamber is 1 mm in height due to its thicker roots (Massalha et al., 2017; Khan et al., 2019). Media and/or inoculation of the microbiome is achieved through additional channels to the main chamber. The PDMS body with the channels is typically bonded on a 50 mm by 75 mm microscope slide, and is made to accommodate multiple plants to increase throughput. Automated control offers the ability for continuous imaging and manipulation of media conditions with high temporal resolution.

One notable example of a microfluidic device for rhizosphere studies is the RootChip, which uses the micro-valves in a PDMS device to control the fluidics (Unger et al., 2000; Grossmann et al., 2011). The first study using the RootChip grew 8 Arabidopsis plants on a single device with micro-valves (Grossmann et al., 2011) but by the second iteration, the throughput has been doubled (Jones et al., 2014; Keinath et al., 2015) indicating rapid technological advances in the field. In addition, all these studies demonstrated spatiotemporal imaging at single-cell resolution and dynamic control of the abiotic environments in the rhizosphere.

Another microfluidics-specific application to rhizosphere study is to use the laminar flow to generate the spatially precise and distinct microenvironment to a section of the root as demonstrated by Meier et al. (2010). A young *Arabidopsis’* seedling was sandwiched and clamped between two layers of PDMS slabs with microchannel features to tightly control synthetic plant hormone flow with 10 to 800 μm resolution to the root tip area, enabling observations of root tissues’ response to the hormones. As many root bacteria produce auxin to stimulate the interactions with the root, this study showed the possible mechanism of microbiome inducing the interaction by stimulating root hair growth. Another application of laminar flow utilized the RootChip architecture by adding the two flanking input channels to generate two co-laminar flows in the root chamber, subjecting a root to two different environmental conditions along the axial direction to study root cells adaptation to the microenvironment at a local level (Stanley et al., 2018). These studies revealed locally asymmetrical growth and gene pattern regulations in Arabidopsis root in response to different environmental stimuli.

Microfluidic platforms have also been successfully employed to study the interactions between the root, microbiome and nematodes in real time (Parashar and Pandey, 2011; Massalha et al., 2017; Aufrecht et al., 2018). In the systems, additional vertical side channels are connected perpendicularly to the main microchannel to enable introduction of microorganisms and solutes to the roots in a spatially and temporally defined manner (Parashar and Pandey, 2011; Aufrecht et al., 2018). A recent microfluidic design incorporated a nano-porous interface which confines the root in place while enabling metabolite sampling from different parts of the root (Patabadige et al., 2019). These studies demonstrated the potential of microfluidics in achieving spatiotemporal insights into the complex interaction networks in the rhizosphere.

Despite several advantages of microfluidics in rhizosphere research as described above, some challenges remain. All the microfluidic applications grow plants in hydroponic systems where clear media is necessary for the imaging applications and packing solid substrates in the micro-channels is not trivial. The microscale of the channels limits the applications of these devices to young seedlings. Thus, interrogating the microscale interactions in bigger, more developed plants is not possible with current microfluidic channel configurations. In addition, technical challenges such as operating the micro-valves and microfabrication present a barrier to device design and construction for non-specialists.

### Fabricated Ecosystem Chambers

Fabricated ecosystems aim to capture critical aspects of ecosystem dynamics within highly controlled laboratory environments (Zengler et al., 2019). They hold promise in accelerating the translation of lab-based studies to field applications and advance science from correlative and observational insights to mechanistic understanding. Pilot scale enclosed ecosystem chambers such as EcoPODs, EcoTrons and EcoCELLs have been developed for such a purpose (Griffin et al., 1996; Lawton, 1996; Ke et al., 2020). These state-of-the-art systems offer the ability to manipulate many parameters such as temperature, humidity, gas composition, etc., to mimic field conditions and are equipped with multiple analytical instruments to link below ground rhizosphere processes to above ground observations and vice versa (Figure 3C; Griffin et al., 1996; Lawton, 1996; Ke et al., 2020). Currently, however, accessibility to such systems is low as there are only several places in the world which can host such multifaceted facilities due to the requirement of significant financial investments.

Switching back to lab-scale systems, a recent perspective paper calls for the need to standardize devices, microbiomes and laboratory techniques to create model ecosystems (Zengler et al., 2019) to enable elucidation of molecular mechanisms mediating observed plant-microbe interactions e.g., exudate driven bacterial recruitment (Zhalnina et al., 2018a, b). Toward this goal, open source 3D printable chambers, termed Ecosystem Fabrication (EcoFAB) devices, have been released with detailed protocols to provide controlled laboratory habitats aimed at promoting mechanistic studies of plant-microbe interactions (Gao et al., 2018). Similar to a rhizotron setup, these flow-through systems are designed to provide clear visual access to the rhizosphere with flexibility of use with either soil or liquid substrates (Figures 3D,E). Certainly, there are many limitations to these devices (discussed in more in Table 1) in that they are limited to relatively small plants and limit the 3D architecture of the root system. Still, an advantage with the EcoFAB is that its 3D printable nature allows for adaptations and modifications to be made and shared on public data platforms such as Github for ease of standardization across different labs and experiments (Sasse et al., 2019). In fact, a recent multi-lab effort showed high reproducibility of root physiological and morphological traits in EcoFAB-grown Brachypodium distachyon plants (Sasse et al., 2019). The development of comparable datasets through the use of standardized systems is crucial to advancing our understanding of complex rhizosphere interactions. Open science programs such as the EcoFAB foster a transparent and collaborative network in an increasingly multi-disciplinary scientific community.

## Perspectives on Current and Future Growth Chamber Designs

Specialized plant chamber systems are necessary for non-destructive visualization of rhizosphere processes and interactions as all destructive sampling approaches tend to overestimate the rhizosphere extent by 3–5 times compared to those based on visualization techniques (Kuzyakov and Razavi, 2019). Nonetheless, plants in such chambers are still grown in defined boundaries and suffer from inherent container impacts. For instance, studies have pointed out that container design (size, density, depth) significantly influences root growth during early developmental stages and leaves lasting impacts on plant health and phenotype (Howell and Harrington, 2004; South et al., 2005; Tsakaldimi et al., 2005; Kostopoulou et al., 2011). The majority of the lab-based chambers are also centimeter scale and are unlikely to replicate exact field conditions in terms of soil structure, water distribution, redox potential or root zone temperatures (Neumann et al., 2009). While comparisons between chamber-grown (e.g., rhizobox) and pot-grown plants show similar outputs (Devienne-Barret et al., 2006), studies comparing plants grown in confined spaces to those directly grown in the field are missing.

A recent review mapped the gradient boundaries for different rhizosphere aspects (physico-chemical gradients, root exudates and microbial communities, etc.) and found that despite the dynamic nature of each trait, the rhizosphere size and shape exist in a quasi-stationary state due to the opposing directions of their formation processes (Kuzyakov and Razavi, 2019). The generalized rhizosphere boundaries were deducted to be within 0.5–4 mm for most rhizosphere processes except for gases (e.g., O_2_) which exceeds > 4 mm and interestingly, they are independent of plant type, root type, age or soil (Kuzyakov and Razavi, 2019). Bearing this in mind, our assessment of the different growth chambers revealed possible overestimation of rhizosphere ranges in some chamber set ups. For instance, the use of root-free soil pouches representing rhizosphere soil despite being cm-distance away from the rhizoplane. This prompts the need for careful evaluation of new growth chamber designs to ensure accurate simulation of natural rhizosphere conditions.

To date, many rhizosphere microbiome studies and growth chambers systems focus on the impact of plant developmental stage, genotype and soil type on microbial composition and function (Chaparro et al., 2014; Edwards et al., 2015; Wagner et al., 2016). On the other hand, predation as a driver in the rhizosphere microbiome remains understudied. For instance, protists are abundant in the soil and are active consumers of bacteria and fungi and play a role in nutrient cycling yet remain an overlooked part of the rhizosphere (Gao et al., 2019). Viruses are also pivotal in modulating host communities thereby affecting biogeochemical cycles but their influence in the rhizosphere is poorly studied (Bi et al., 2020). These predator-prey interactions in the rhizosphere deserve in-depth studies which can be facilitated by these specialized growth chambers.

Another area worth investigating in the rhizosphere is in anaerobic microbial ecology. At microbially relevant scales, soils primarily exist as aggregates (<2 mm). Aggregation creates conditions different from bulk soil, particularly in terms of oxygen diffusion and water flow resulting in anoxic spaces within aggregates and influences the microbial community (Wilpiszeski et al., 2019). The rhizosphere is also rich in a wide range of compounds which can serve as alternative electron acceptors such as nitrate, iron, sulfate and humic substances in the absence of oxygen (Lecomte et al., 2018). However, most anaerobic studies in the rhizosphere focus only on aqueous environments such as water-logged paddy soils despite biochemical and metatranscriptomic evidence pointing to the possibility of anaerobic respiration in the rhizosphere (Lecomte et al., 2018). To fully understand biogeochemical cycles in the rhizosphere, it is imperative to investigate rhizosphere processes in the microscale and to include localized redox conditions as one of the influencing parameters. Microfluidic platforms with its fast prototyping capabilities can be helpful in creating growth chambers designed to stimulate these redox changes.

In the study of the rhizosphere microbiome, genetic manipulation strategies are foundational in deep characterization of microbial mechanisms and current manipulation techniques require axenic isolates. However, the uncultivability of a significant portion of soil microorganisms continues to hamper efforts in gaining mechanistic knowledge. Even for culturable isolates, the process of isolation introduces selective pressure and disturbance to the community with inevitable loss of information on spatial interactions. A recent innovation in gene editing technologies using CRISPR-cas systems demonstrated *in situ* editing of genetically tractable bacteria within a complex community (Rubin et al., 2020). Coupled with the use of transparent soil-like substrates (Downie et al., 2014), the application of such a technique for the editing of *in situ* rhizosphere microbiome while preserving spatial and temporal associations would indeed bring invaluable insights. Specialized growth chambers using 3D fabrication and microfluidic technologies are primed to facilitate such innovations.

Finally, this review revealed that while similarities exist among the different growth chamber systems, many of these systems are bespoke. This makes it difficult to replicate experiments and determine reproducibility which are important cornerstones of scientific advancement. The complexity of rhizosphere interactions also warrant that computational models are essential to gain a better understanding of system level processes (Darrah et al., 2006; Zengler et al., 2019). However, predictive modeling requires data from standardized approaches to be comparable between experiments. Thus, future growth chamber systems and designs are encouraged to follow the open science framework to enable standardization to an extent, such as in the case of EcoFABs (Sasse et al., 2019).

## Conclusion

Studying the rhizosphere is a challenge due to the complex and dynamic interactions between many of its members, made further complicated by the opaque soil. Specialized plant chambers have been and continue to be an important tool in investigating these rhizosphere spatiotemporal processes in the soil. We identified representative growth systems used to study various rhizosphere interactions and processes such as root system architecture, exudation and microbial communities and found that they share common features but most are custom made to answer specific research questions. A major benefit of these specialized chambers is the ability to visualize the rhizosphere which allows for coupling with various analytical instruments to probe *in situ* processes through non-destructive sampling. Modern developments in growth chamber systems utilizing 3D fabrication and microfluidic technologies are also gaining prominence in understanding microscale interactions. These chambers also present the opportunity for both top down (community engineering and characterization) and bottom up (isolation-based) approaches to investigate rhizosphere communities. However, it should be noted that as these specialized chambers have been developed for model systems, the findings should ultimately be verified at field relevant conditions for truly predictive ecological understanding. Nonetheless, it is clear that the use of specialized chambers would continue to play a central role in our effort to gain a mechanistic understanding of the rhizosphere ecosystem.

## Author Contributions

RC conceptualized the idea. MY developed and wrote the manuscript. PK and YL contributed to specific sections of the review. All authors contributed to drafting and editing of the manuscript.

## Conflict of Interest

The authors declare that the research was conducted in the absence of any commercial or financial relationships that could be construed as a potential conflict of interest.
